# BOP1 contributes to the activation of autophagy in polycystic ovary syndrome via nucleolar stress response

**DOI:** 10.1007/s00018-023-05091-1

**Published:** 2024-02-27

**Authors:** Rui Ji, Zhimo Zhang, Zhe Yang, Xin Chen, Tailang Yin, Jing Yang

**Affiliations:** 1 Reproductive Medical Center, Renmin Hospital of Wuhan University, Wuhan, China; 2 Hubei Clinic Research Center for Assisted Reproductive Technology and Embryonic Development, Wuhan, China

**Keywords:** Polycystic ovary syndrome (PCOS), Block of proliferation 1 (BOP1), Autophagy, Nucleolus stress

## Abstract

Abnormal autophagy is one of the vital features in polycystic ovary syndrome (PCOS). However, the underlying molecular mechanisms remain unelucidated. In this study, we aimed to investigate whether Block of Proliferation 1 (BOP1) is involved in the onset of autophagy activation of granulosa cells in PCOS. Firstly, we found that BOP1 expression was significantly down-regulated in the ovaries of PCOS mice, which was associated with the development of PCOS. Next, local injection of lentiviral vectors in the ovary for the overexpression of BOP1 significantly alleviated the phenotypes of elevated androgens, disturbed estrous cycle, and abnormal follicular development in PCOS mice. Subsequently, we found that knockdown of BOP1 activated autophagy of granulosa cells in the in vitro experiments, whereas overexpression of BOP1 inhibited autophagy in both in vivo and in vitro models. Mechanistically, BOP1 knockdown triggered the nucleolus stress response, which caused RPL11 to be released from the nucleolus into the nucleoplasm and inhibited the E3 ubiquitination ligase of MDM2, thereby enhancing the stability of p53. Subsequently, P53 inhibited mTOR, thereby activating autophagy in granulosa cells. In addition, the mRNA level of BOP1 was negatively correlated with antral follicle count (AFC), body–mass index (BMI), serum androgen levels, and anti-Mullerian hormone (AMH) in patients with PCOS. In summary, our study demonstrates that BOP1 downregulation inhibits mTOR phosphorylation through activation of the p53-dependent nucleolus stress response, which subsequently contributes to aberrant autophagy in granulosa cells, revealing that BOP1 may be a key target for probing the mechanisms of PCOS.

**Supplementary Information:**

The online version contains supplementary material available at 10.1007/s00018-023-05091-1.

## Introduction

Polycystic ovary syndrome (PCOS) is a reproductive endocrine disorder that affects 5%–18% of women of childbearing age worldwide [1]. The clinical manifestations of PCOS include hyperandrogenism and oligoovulation, which are generally accompanied by metabolic abnormalities such as insulin resistance (IR), obesity, and cardiovascular diseases [2–4]. Nevertheless, owing to the heterogeneity of the condition and the absence of a well-defined pathogenic mechanism, at present, research and treatment achievements for PCOS are relatively limited.

Autophagy is a specific type of programmed cell death involving the delivery of “cargo,” including organelles, to the lysosome for degradation [5]. To the best of our knowledge, there are three main types of autophagy: macroautophagy, chaperone-mediated autophagy, and microautophagy [6]. Among them, macroautophagy is the main mechanism underlying molecular degradation and reuse in eukaryotic organisms to maintain nutrient and energy homeostasis in cells [7]. Recent studies have reported that autophagy is involved in PCOS onset and development and that its abnormal activation or inhibition affects follicular development and promotes ovulation disorders. Furthermore, autophagy may be involved in the development of hyperandrogenemia and metabolic abnormalities in individuals with PCOS [8]. In PCOS, autophagy status varies based on the cell type [9]. For example, autophagy is abnormally activated in ovarian granulosa cells (GCs) in patients with PCOS. GCs grow around the oocyte and promote its growth and maturation by exchanging nutrients and molecular signals. The activation of the autophagy of GCs decreases the outer GC layer of the oocyte, thereby affecting sex hormone secretion and preventing oocyte maturation; this leads to sporadic ovulation in women with PCOS, affecting their fertility potential [10].

Block of proliferation 1 (BOP1), a member of the PES1–BOP1–WDR12 (PeBoW) complex, is a highly conserved ribonucleoprotein. Studies have reported that it is involved in 28S and 5.8S ribosomal RNA (rRNA) processing and 60S ribosomal biosynthesis [11]. BOP1 downregulation or mutation affects rRNA synthesis and ribosome biogenesis and may lead to p53-associated cell cycle arrest [12]. Owing to the vital role of BOP1 in cellular biosynthesis, it has been comprehensively evaluated in studies on drug resistance in cancer and the mechanisms underlying tumorigenesis [13, 14]. In studies on female tumors, BOP1 downregulation inhibited drug resistance in triple-negative breast cancer [15]. Furthermore, another study confirmed that BOP1 was significantly downregulated at the RNA level when follicular development and differentiation were inhibited via genetic intervention in hens; this suggests that it plays an underlying role in GC proliferation and differentiation and follicular development [16]. However, studies on the role of BOP1 in PCOS are lacking.

Ribosome biogenesis is a complex multistep process with most of its steps occurring in the nucleolus [17]. The nucleolus is a dynamic membrane-free organelle localized in the nucleus. It is divided into three compartments: fibrous center, dense center and granular component. Ribosome biogenesis begins in the FC. The RNA polymerase I transcription machinery transcribes rDNA into the 47S rRNA; thereafter, the 47rRNA is processed and cleaved in the DFC and GC to produce mature 18S, 5.8S, and 28S rRNAs. Abnormalities in any steps of ribosome biosynthesis may result in morphological and functional alterations in the nucleolus, a process called nucleolar stress [18]. In nucleolar stress responses, some ribosomal proteins are translocated from the nucleolus to the nucleoplasm [19]; these proteins activate signaling pathways, including p53, thereby affecting cellular homeostasis [20]. It has been found that adverse stimuli in the external environment and mutations in genes in certain diseases may impair ribosomal biosynthesis, which in turn leads to nucleolar stress [17]. Therefore, alterations in BOP1, an important factor in ribosome biogenesis, during the disease process may promote nucleolar stress, which in turn affects normal physiological processes of the cells.

In the present study, using database analysis, we identified the effects of BOP1 downregulation in a PCOS disease model and elucidated the novel finding that BOP1 activates p53-dependent nucleolus stress responses and ultimately regulates the autophagy of GCs in PCOS.

## Material and methods

### Clinical sample

Human ovarian GCs were obtained from 20 patients with PCOS and 20 healthy control women undergoing in vitro fertilization and embryo transfer (IVF-ET) at Renmin Hospital of Wuhan University. This study obtained approval from the Ethics Committee of Renmin Hospital of Wuhan University (No. WDRY2019-K077). The participants signed informed consent forms. The diagnostic criteria for PCOS were in accordance with the 2003 Rotterdam criteria. Supplementary Table 1 summarizes the clinical characteristics of the patients who participated in this study. Human ovarian GCs were obtained from patients as follicular fluid as mentioned previously [21].

### Cell culture and transfection

KGN, a human GC line, Obtained from ATCC, was cultured in DMEM/F12 medium (Gibco, Grand Island, NE, United States) supplemented with 10% fetal bovine serum (Gibco) and 100 μg/mL antibiotics (a mixture of penicillin and streptomycin; Welgene) at 37 °C in a 5% CO_2_ incubator.

The cells were treated with 10 μM of PFT-α (Selleck, USA) or equal dimethyl sulfoxide (DMSO; Servicebio, Wuhan, China) for 12 h and then the cells were digested with enzyme and collected for Western blotting. The cells were subjected to starvation for the indicated periods using EBSS (Procell, Wuhan, China) and detected via autophagy flux using 10 μM of chloroquine (CQ, MedChemExpress, Shanghai, China). Furthermore, the cells were treated with 50 of μg/mL cycloheximide (CHX, Selleck, State of *Texas*, USA) and collected every 30 min to determine the stability of the p53 protein.

### Lentivirus infection and RNA interference

For the knockdown of the BOP1 expression, KGN cells in the logarithmic growth were infected with BOP1 shRNA lentivirus provided by GeneChem BioTECH (Shanghai, China) in the logarithmic growth phase. Lentivirus-loading BOP1 RNAi sequence contained RNAi-1: GATAGCAAGCTGGTGTGGTTT, RNAi-2: CGCCACAAGATGCACGTACCT, and RNAi-3: TGGAGTGGTACGATGACTT. The negative control shRNA sequence was TTCTCCGAACGTGTCACGT.

For the overexpression of BOP1, KGN cells were infected using BOP1 expression lentivirus vector and blank vector (GeneChem BioTECH, Shanghai, China). After infection, the cells were cultured for 72 h, and then stable cell lines were screened with puromycin.

For the detection of autophagy flux, the KGN cells were infected with adenoviral vectors encoding MRFP-GFP-LC3 (Hanbio Biotechnology, Shanghai, China). After the infection, the cells were cultured for 48 h and the LC3 fluorescent spots were visualized with a confocal microscope (Olympus FV1000) after appropriate treatment.

For the knockdown RPL11 expression, RPL11 siRNA (sequence: GGAACUUCGCAUCCGCAAATT, Genepharma, Shanghai, China) was transfected into KGN cells with Lipofectamine 2000 (Invitrogen, USA).

### Animal model

Prepubescent Sprague–Dawley rats (*n* = 12), aged 21 days kept in clean cages on a 12-h light–dark cycle, were divided randomly into the following two groups: the control and the dehydroepiandrosterone (DHEA) group (*n* = 6 in each group). Rats in the DHEA group received subcutaneous injections of DHEA (6 mg/100 g body weight, dissolved in corn oil) (MedChemExpress), whereas rats in the control group received corn oil. The rats underwent treatment in the constant order for 21 days.

C57BL/6 female mice, aged 21 days, were used for the subsequent experiments (*n* = 24). To elucidate the mechanism of BOP1 underlying the development of PCOS, the BOP1 expression lentivirus and blank vector were mixed in F-127 gel (Sigma, Burlington, Massachusetts, USA) and supplemented with 0.25% trypsin (Servicebio) and added onto the bilateral ovaries of the mice. After 5 days, the mice in the DHEA group (DHEA + Vector group and DHEA + BOP1 group) were treated with DHEA (6 mg/100 g body weight, dissolved in corn oil) to establish the PCOS model as described in our previous study [22]. Mice in the control group (Control + Vector group and Control + BOP1 group) were treated with corn oil only (*n* = 6 in each group). The weight of mice was recorded continuously throughout the study period. The mice underwent treatment in the constant order for 21 days.

After the 21-day modeling, all mice underwent fasting for 16 h. Blood was obtained from the eye veins of the mice. The mice were killed and both ovaries were removed. Some parts of the ovaries were sliced and embedded on paraffin wax, and the remaining part was stored in a refrigerator at − 80 °C until further experiments.

All experiments on the animals were designed according to the Guide for the Care and Use of Laboratory Animals. All animal investigations were conducted at Wuhan University's Renmin Hospital’s Cardiovascular Surgery Laboratory.

### Estrous cycle analysis

From the 11th to the 21st day of treatment, vaginal smears were taken every day at 08:30 a.m. The estrous cycle stage was identified using the principal cell types in vaginal smears, which were stained by hematoxylin and eosin (HE) and observed using an optical microscope.

### Evaluation of serum testosterone levels

Venous blood was kept at room temperature for 1 h and then centrifuged for 20 min at 2500 rpm for the serum. The serum testosterone levels were detected using a Testosterone ELISA kit (Xinfan Biotech, Shanghai, China).

### HE staining of ovaries

Staining was performed following a previously described protocol [22].

### RNA extraction and reverse transcription polymerase chain reaction (PCR) analysis

Total RNA from the GCs was extracted using RNAex Pro Reagent (Accurate Biotech) and reverse transcribed according to the manufacturer’s instructions. PCR was performed on a LightCycler 480 II instrument using SYBR Green Premix Pro Taq HS qPCR Kit (Accurate Biotech). Primer sequences used in this experiment are listed in Supplementary Table 2. Data were calculated using the 2^–△△Ct^ method.

### Molecular docking

The HDOCK online platform (http://hdock.phys.hust.edu.cn/) was used to study the molecular docking mechanism. Furthermore, the UniProt protein database was used to obtain the structures of the docking proteins RPL11 (ID: P62913) and BOP1 (ID: Q14137). Additionally, the PyMOL (version 4.3.0) software was used to separate original ligands and protein structures and dehydrate and eliminate organic debris. The Ligplus software was used to calculate the force between the two proteins at a two-dimensional angle. The interacting amino acid residues of the two proteins were then mapped using PyMOL (version 4.3.0).

### Western blotting

Protein extraction and Western blotting were performed, as described previously [22]. Equal amounts of protein samples were separated via SDS-PAGE gels and transferred onto PVDF membranes (Millipore, USA). The membranes were blocked with 5% dried skim milk for 1 h and then incubated with primary antibodies: BOP1[(Abcam, Rabbit, ab252819, 1:5000,used for human tissue and KGN cells), (Bioss, Rabbit, ab252819,1:1000,used for tissue of mice and mouse)], LC3B (Abcam, Rabbit, ab192890, 1:2000), SQSTM1 (Proteintech, Rabbit, 18420-1-AP 1:5000), Beclin1 (Proteintech, Rabbit, 11306-1-AP, 1:1000), RPL11 (Abcam, Rabbit, ab79352,1:1000), MDM2 (Santa Cruz, mouse, sc-965, 1:500), p53 (Proteintech, Rabbit, 60283-2-Ig, 1:5000), mTOR(Cell Signaling, Rabbit, 2972, 1:1000), p-mTOR (Cell Signaling, Rabbit, 2971, 1:1000), ERK(Proteintech, Rabbit, 11-257-1-AP), p-ERK (Cell Signaling, Rabbit, 4370, 1:2000), AMPK (Abcam, Rabbit, ab133448, 1:1000), p-AMPK (Cell Signaling, Rabbit, ab80039, 1:1000), AKT (Proteintech, mouse, 60203-2-Ig 1:1000), p-AKT (Proteintech, Rabbit, 80455-1-RR, 1:2000), and β-actin (Proteintech, Rabbit, 81115-1-RR 1:5000). The membrane was incubated with the primary antibodies for overnight at 4 °C and then rinsed thrice with TBST before treating with HRP conjugated Rabbit Anti-Goat IgG (H + L) or HRP conjugated Goat Anti-Mouse IgG (H + L) for 1 h. The bands were visualized using the Bio-Rad ChemiDocTM XRS + System.

### Co-immunoprecipitation (CO-IP)

Proteins were extracted using the KGN cell lysis buffer and subjected to western blotting (Beyotime, Shanghai, China). Briefly, the cell lysates were centrifuged at 12,000 rpm for 20 min at 4 °C, and the supernatants were mixed with 5 μL of primary antibodies and 40 μL of protein A + G agarose suspension beads (Santa Cruz, Dallas, Texas, USA) to incubate overnight at 4 °C. The beads were rinsed with a lysis buffer the following day. Next, the samples were boiled in a loading buffer for 10 min at 100 °C before subjecting them to western blotting.

KGN cells were transfected using the His-Ub plasmid and then treated with 30 μM MG132, (Sigma-Aldrich) for 6 h. Subsequently, a portion of the lysates was incubated with anti-His monoclonal antibody (Cell signaling, Rabbit, 2365, 1:1000) and used for ubiquitylation experiments; bead-binding proteins and the rest of the lysates were analyzed with IB.

### Immunostaining

KGN cells were fixed with 4% paraformaldehyde and then treated with 10% goat serum for 30 min to allow for cell immunofluorescence. Then, they were treated with anti-RPL11 and anti-MDM2 antibodies (1:50) at 4 ℃. On the second day, the cells were rinsed with PBST and then incubated with secondary an Cy3 conjugated Goat Anti-Rabbit IgG (H + L) and FITC conjugated Goat Anti-Mouse IgG (H + L) for 1 h. The nuclei were stained with 4',6-diamidino-2-phenylindole for 5 min. Next, the cells were visualized using a confocal microscope.

To stop endogenous peroxidase activity, the paraffin sections were boiled in an antigen retrieval solution in a microwave oven, followed by treatment with 0.3% hydrogen peroxide for 10 min. After 30 min, the sections were incubated with 10% normal goat serum and treated with anti-BOP1 (1:50), anti-LC3 (1:100), and anti-SQSTM1 (1:100) antibodies overnight. The paraffin sections were then washed thrice with PBST, followed by treatment with secondary antibodies for 2 h. The sections were then stained with hematoxylin and submerged in 3,3-diaminobenzidine (Servicebio).

### Statistical analysis

Statistical analyses were performed using GraphPad Prism 9. The statistical significance of the two groups was determined by Student’s two-tailed t-test, whereas multiple comparisons were analyzed by one-way analysis of variance, followed by Tukey’s post-hoc test. A value of *P* < 0.05 was considered significant.

## Results

### BOP1 is downregulated in patients with PCOS and animal models

**Fig. 1:**
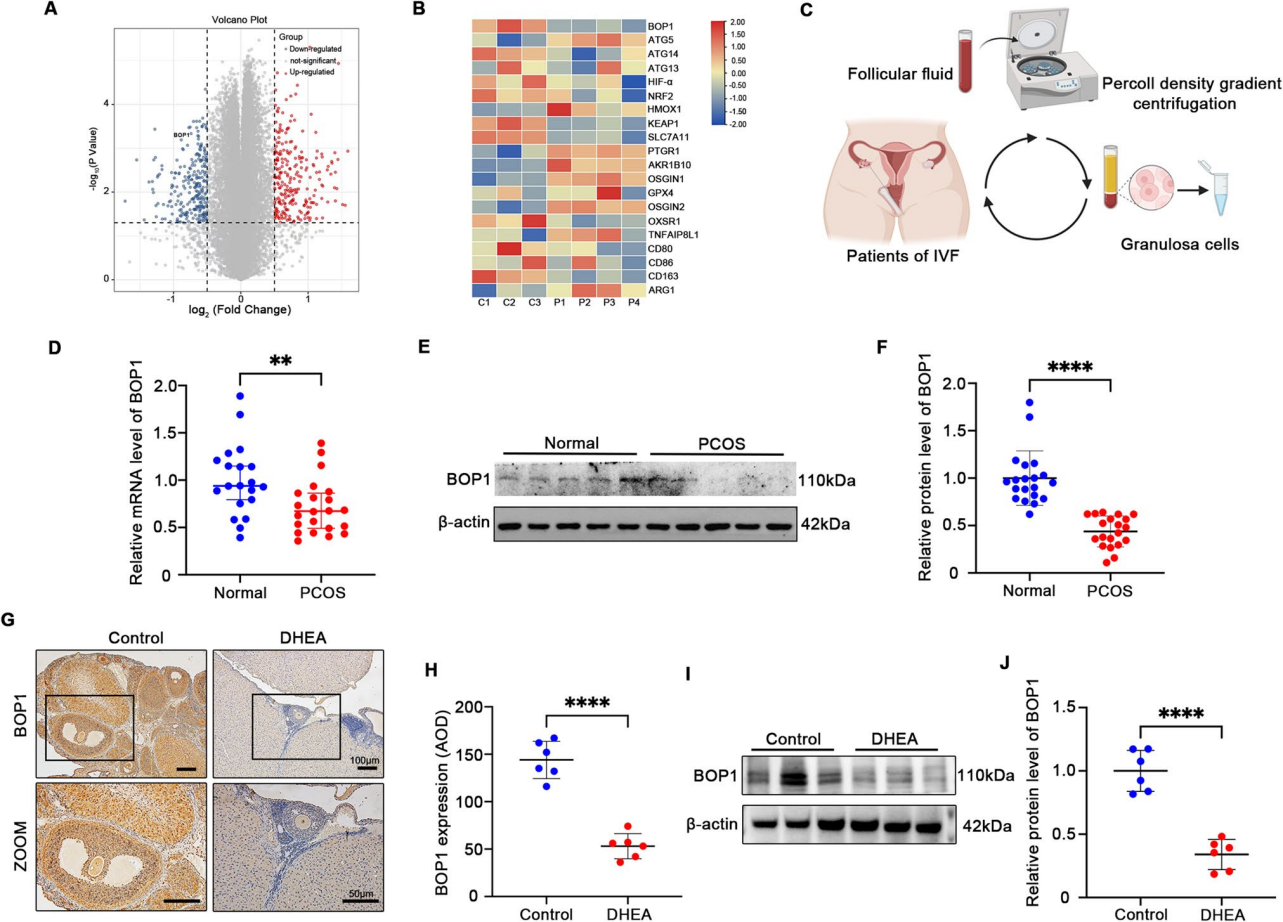
BOP1 is downregulated in PCOS patients and animal models. **A** Volcano plot of gene expression changes in the PCOS ovaries (*n* = 4) versus control (*n* = 3). **B** Heatmap of autophagy related genes, oxidative stress molecules and inflammatory cytokine expression in the DEGs. **C** Diagram of human granulosa cell extraction process. **D** Quantification of the mRNA level of BOP1 in PCOS patients and healthy control women (*n* = 20). **E**, **F** Quantification of the protein level of BOP1 in PCOS patients and healthy control women (*n* = 20). **G**, **H**. Immunohistochemical analysis of BOP1 expression in PCOS Rats with controls (*n* = 6) **I**, **J**. Western blot and densitometric analysis for the expression of BOP1 in DHEA-treated rats and controls. (*n* = 6). Data are presented as mean ± SD. Student’s *t* test. **p < 0.01; ****p < 0.0001; *ns* not significant

To analyze potential genes that may affect PCOS, we used the GEO dataset (GEO ID: GSE148839) to obtain differentially expressed genes (DEGs) from the ovaries of the control and PCOS mice, and 102 DEGs were obtained (Fig. 1A). Among them, genes associated with inflammation, autophagy, and oxidative stress differed between the ovaries of the PCOS and control mice (Fig. 1B), which is consistent with previous results. Among the 102 DEGs, bop1 was the gene of interest. It was significantly downregulated in the ovaries of the PCOS mice compared with that in the control group (Fig. 1A, B). Subsequently, we collected follicular fluid from patients with PCOS and healthy controls undergoing IVF-ET. The fluid was subjected to density gradient centrifugation to isolate and obtain GCs, and then BOP1 mRNA levels in these isolated GCs were measured (Fig. 1C). The results revealed decreased BOP1 mRNA levels in the GCs of the patients (Fig. 1D). Thus, we measured BOP1 protein levels in the GCs of the patients, and the results were similar to the mRNA detection results (Fig. 1E, F). To explore the BOP1 expression in PCOS animals, we modeled rats using DHEA and examined BOP1 levels in their ovaries. The immunohistochemical results showed that BOP1 expression was significantly lower in the PCOS rats than in the controls (Fig. 1G, H). The western blotting results of extracted ovarian tissues revealed that BOP1 protein levels decreased significantly in the PCOS group (Fig. 1I, J). In summary, BOP1 expression was downregulated in patients with PCOS and animal models.

### BOP1 overexpression ameliorates hyperandrogenism, estrous cycle disruption, and follicular developmental abnormalities in PCOS mice

**Fig. 2:**
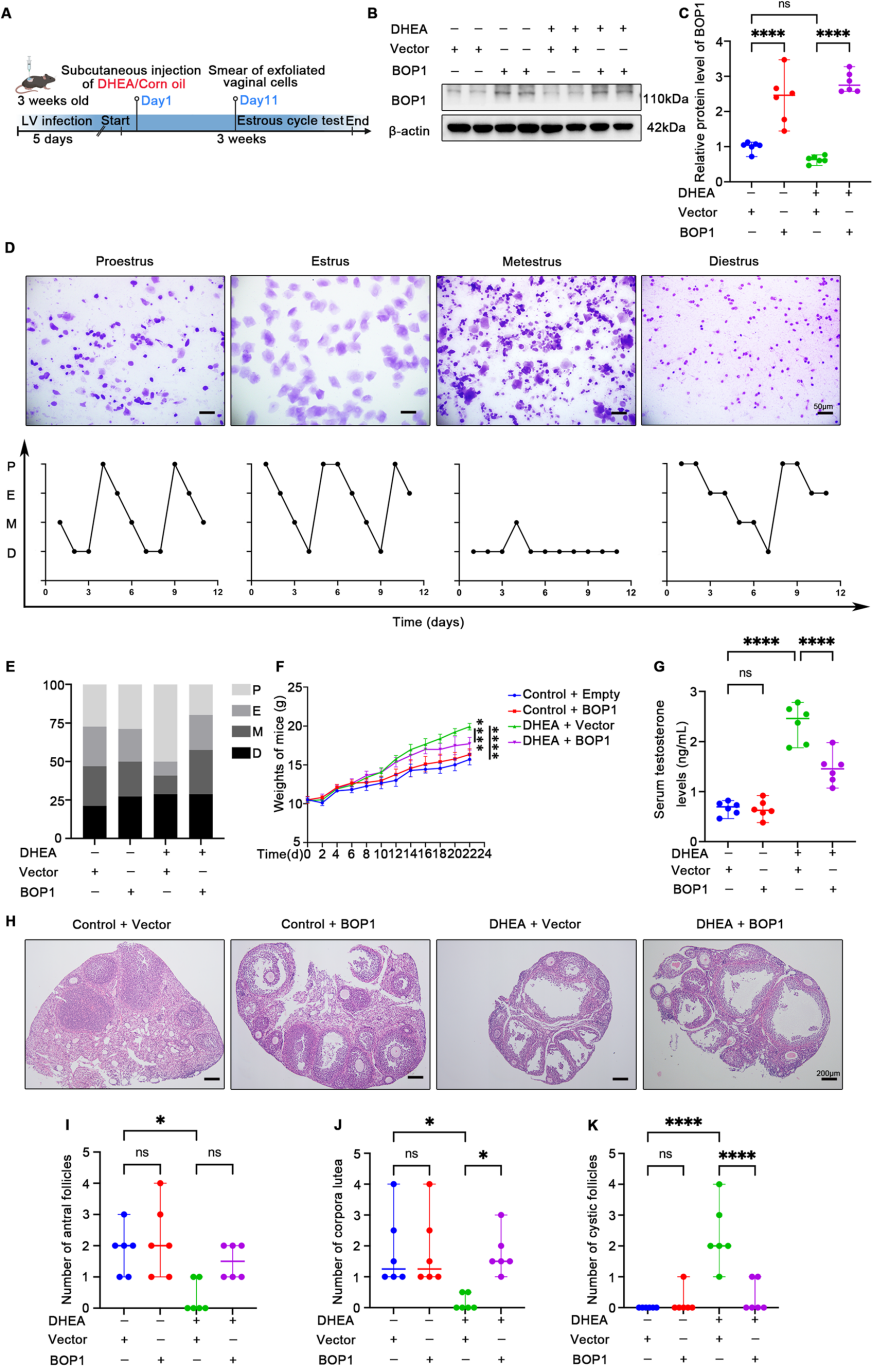
BOP1 overexpression ameliorates abnormal phenotype in PCOS mice. **A** Pattern of PCOS modeling after local infection of mouse ovaries using BOP1 overexpression lentivirus or vector lentivirus. **B**, **C** Western blot and densitometric analysis for the expression of BOP1 in lentivirus-infected PCOS mice and controls (*n* = 6). **D**, **E** Assessment of the estrous cycle in mice (*n* = 6). *P* proestrus, *E* Estrus, *M* metestrus, *D* diestrus. **F**. Body weight measurements in mice (*n* = 6). **G** Detection of serum testosterone level in mice (*n* = 6). **H** Representative H&E staining images of ovarian morphology in the four groups of mice. **I**–**K**. Number of each follicle in four groups of mice was shown (*n* = 6). Data are presented as mean ± SD. one-way ANOVA. *p < 0.05; ****p < 0.0001; *ns* not significant

Based on *bop1* downregulation in patients and animals with PCOS, we speculated that reversing its expression would improve PCOS-related phenotypes. Therefore, a BOP1 overexpression vector or an empty lentivirus conjugated with 0.25% trypsinized F-127 was introduced into the ovaries of three-week-old C57BL6 female mice, followed by PCOS modeling over 21 days (Fig. 2A). The western blotting results showed that ovarian BOP1 levels were higher in the BOP1 overexpression group than in the vector lentivirus group of the mice (Fig. 2B, C). We found that compared with the control mice, the PCOS mice showed a significantly disrupted estrous cycle and a prolonged diestrus period; however, the disrupted estrous cycle improved in the PCOS mice treated with LV-BOP1 (Fig. 2D, E). During the 21 days of modeling, the PCOS mice gained significantly more weight than the controls, and LV-BOP1 treatment alleviated the excessive weight gain to some extent (Fig. 2F).

To investigate whether BOP1 overexpression affected the hyperandrogenic phenotype of the PCOS mice, we examined the androgen levels in mouse serum. The results showed that androgen levels were significantly higher in the DHEA group than in the control group of the mice, whereas the LV-BOP1 intervention partially lowered the increased androgen levels (Fig. 2G). The ovary morphology showed that the number of antral follicles and corpus luteum was lower and the number of cystic follicles was higher in the ovaries of the PCOS mice than in the control group, whereas the abnormalities of follicular development in the PCOS mice treated with LV-BOP1 were attenuated compared with those in the PCOS mice treated with the vector lentivirus (Fig. 2H–K). Overall, the above data suggest that BOP1 overexpression reverses the phenotypes of the disturbed estrous cycle, excessive weight gain, hyperandrogenemia, and abnormal follicular development in PCOS mice.

### BOP1 is involved in autophagy regulation in PCOS

**Fig. 3:**
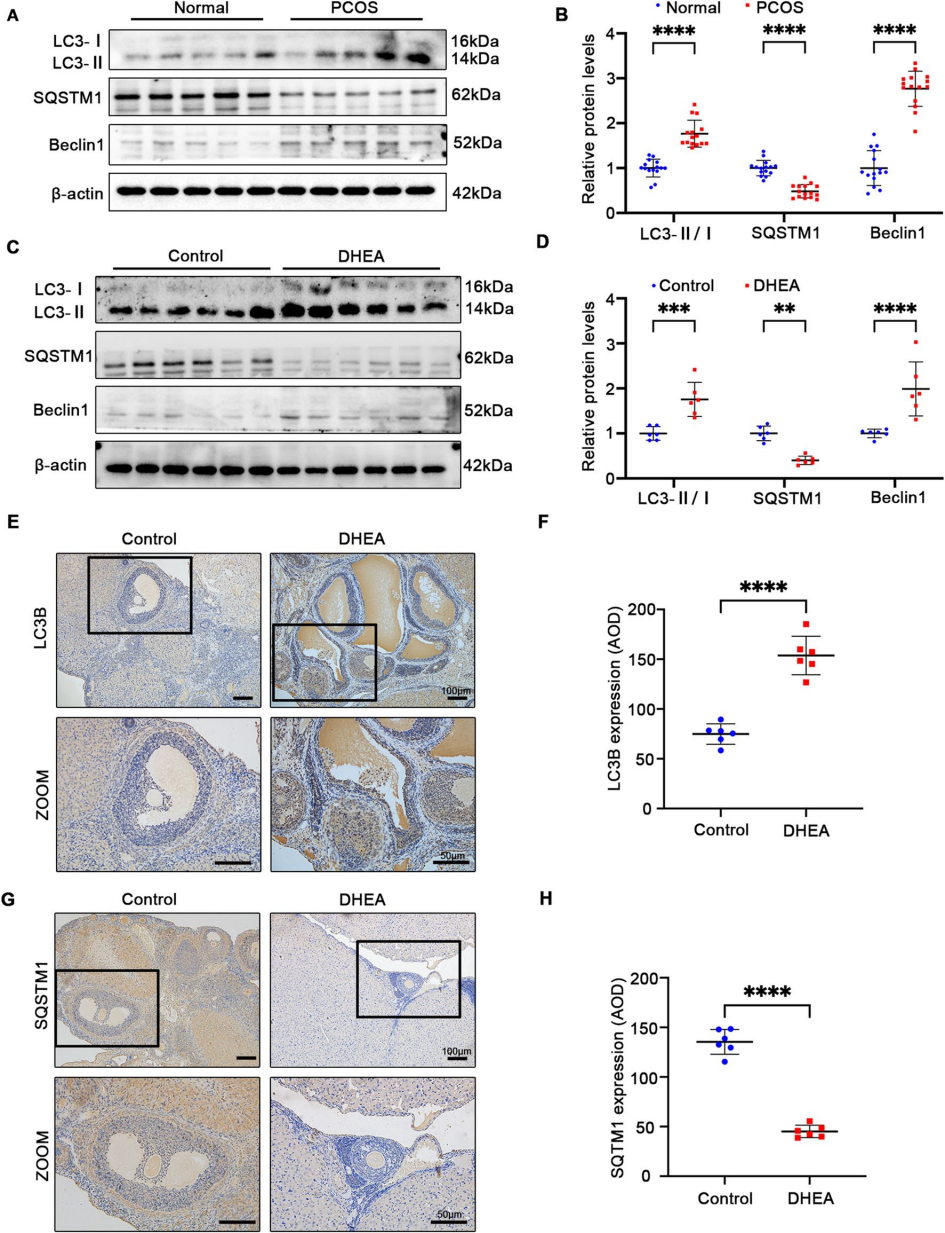
Autophagy is activated in PCOS patients and rats. **A**, **B** Western blot and densitometric analysis for the expression of LC3B, SQSTM1, Beclin1 in PCOS patients and normal group (*n* = 15). **C** D. Western blot and densitometric analysis for the expression of LC3B, SQSTM1, Beclin1 in DHEA-treated rats and controls (*n* = 6). E, F. Immunohistochemical analysis of LC3B expression in PCOS Rats with controls (*n* = 6). **G**, **H** Immunohistochemical analysis of SQSTM1 expression in PCOS Rats with controls (*n* = 6). Data are presented as mean ± SD. Student’s *t*-test. **p < 0.01; ***p < 0.001; ****p < 0.0001; *ns* not significant. Data are presented as mean ± SD. one-way ANOVA. *p < 0.05; ****p < 0.0001; *ns* not significant

**Fig. 4:**
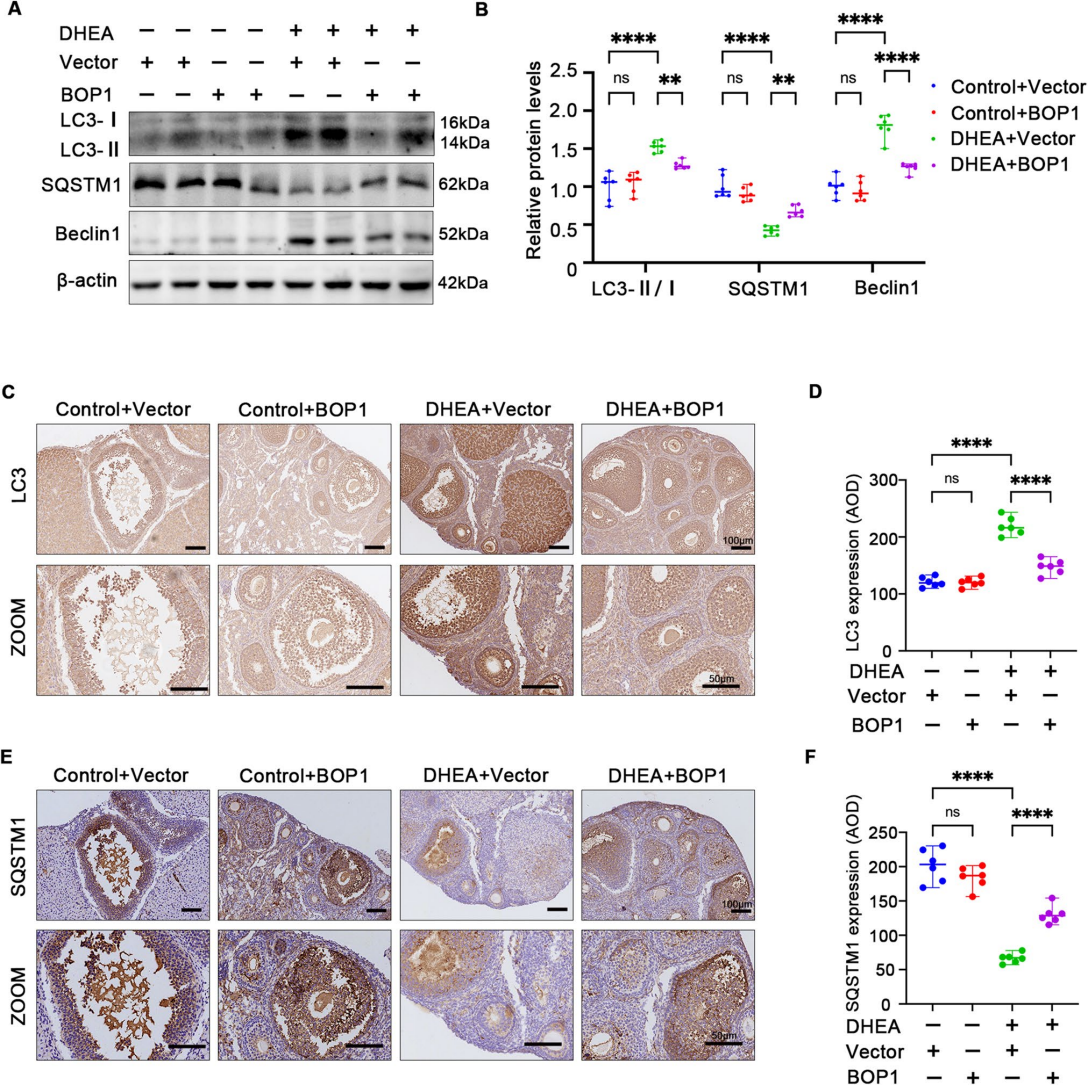
BOP1 overexpression partially alleviated autophagy in PCOS mice. **A**, **B** Western blot and densitometric analysis for the expression of LC3B, SQSTM1, Beclin1 in ovaries of mice in the four groups (*n* = 6). **C**, **D** Immunohistochemical analysis of LC3B expression in the four groups (*n* = 6). **E**, **F** Immunohistochemical analysis of SQSTM1 expression in the four groups (*n* = 6). Data are presented as mean ± SD. one-way ANOVA. **p < 0.01; ****p < 0.0001; *ns* not significant

Previous studies have shown that abnormal autophagy activation in GCs contributes to PCOS development [23]. Thus, we first examined LC3B, Beclin1, and SQSTM1 levels in patients with PCOS and found that compared with the normal group, the patients with PCOS showed an increased LC3-II/LC3-I ratio and Beclin1 levels and decreased SQSTM1 levels in GCs (Fig. 3A, B), suggesting that autophagy was activated in the GCs of patients with PCOS. Additionally, the western blotting of the ovarian tissues from PCOS rats showed an increased LC3-II/LC3-I ratio and Beclin1 levels and decreased SQSTM1 levels (Fig. 3C, D). Consistently, the immunohistochemical analysis of the ovarian tissues suggested autophagy activation in the PCOS rats (Fig. 3E–H). Further, we explored autophagy alteration in the PCOS mice. The western blotting results showed an increased LC3-II/LC3-I ratio and Beclin1 levels and decreased SQSTM1 levels in the ovarian tissues of the PCOS mice compared with those in the control mice, suggesting autophagy activation in the PCOS mice, whereas the autophagy level in the PCOS mice treated with LV-BOP1 decreased partially (Fig. 4A, B). The immunohistochemical results showed increased LC3B levels and decreased SQSTM1 levels in the ovarian GCs of the PCOS mice, whereas these alterations were partially reversed in the GCs of the BOP1-overexpressing PCOS mice (Fig. 4C–F). The above results suggest that BOP1 may regulate autophagy levels in PCOS mice.

### Downregulation of BOP1 activates autophagy in KGN

**Fig. 5:**
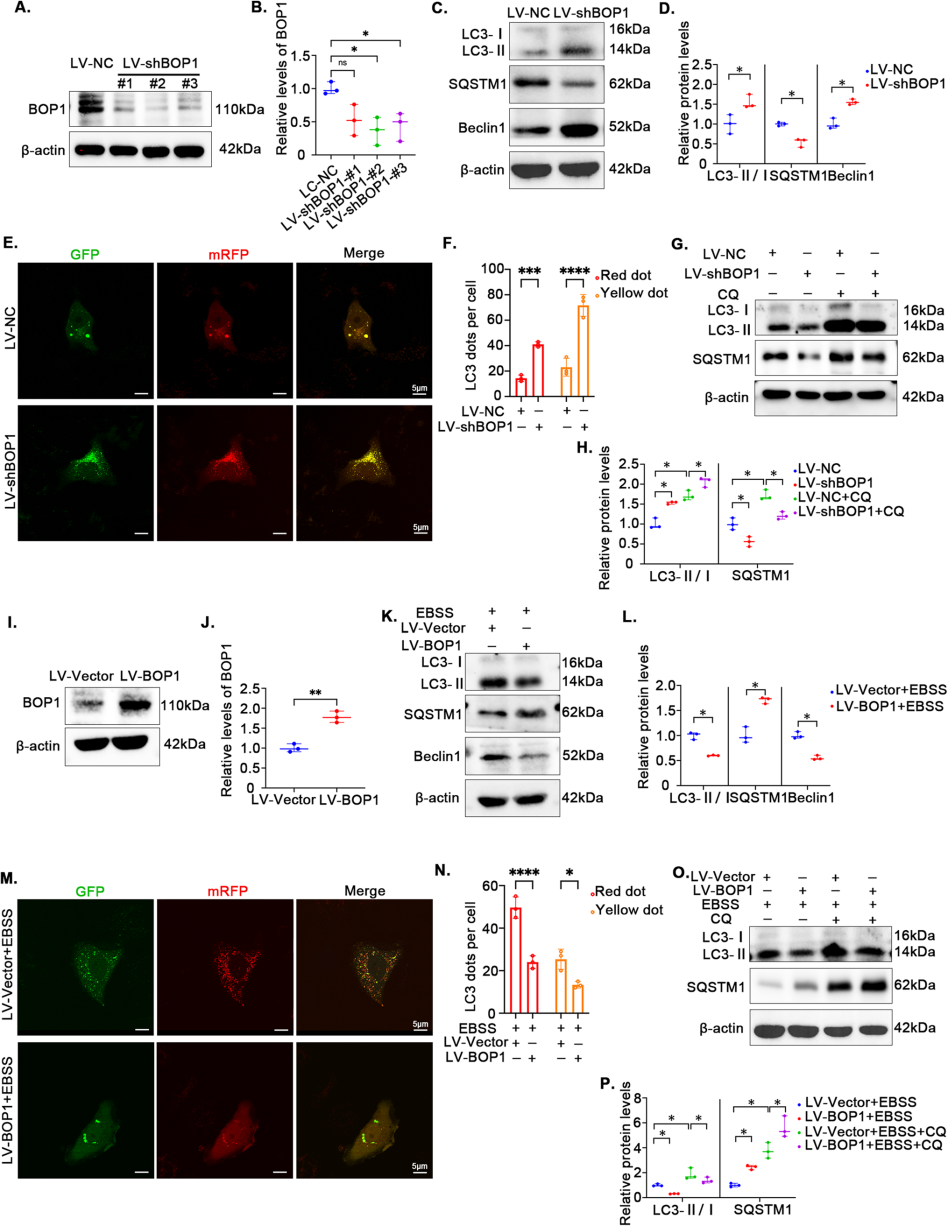
Downregulation of BOP1 activates autophagy in KGN. **A**, **B** Representative western blotting bands and the relative expression levels of BOP1 in KGN cells infected with LV-NC or LV-shBOP1. *n* = 3 for each group. *p < 0.05, significantly different from LV-NC, Student’s *t* test. **C**, **D** Western blot and densitometric analysis for the expression of LC3B, SQSTM1, Beclin1 in KGN cells (*n* = 3). **E**, **F** KGN cells were transfected with mRFP-GFP-LC3 recombinant adenovirus for detection of autophagy flux. Representative images of autophagosomes (yellow spots) and autolysosomes (red spots). Student’s *t* test. G, H. KGN cells were treated with 10 μM CQ or DMSO for 4 h. Representative western blotting bands and the relative expression levels of LC3B and SQSTM1 were detected. (*n* = 3). one-way ANOVA. **I**, **J** Representative western blotting bands and the relative expression levels of BOP1 in KGN cells infected with LV-Vector or LV-BOP1. *n* = 3 for each group. Student’s *t* test. **K**, **L**. KGN cells were subjected to starvation for the using EBSS for 4 h. Quantification of the protein level of LC3B, SQSTM1 and Beclin1 were detected. (*n* = 3). Student’s *t* test. **M**, **N** Autophagic flux was assessed by quantification of autophagosomes (yellow spots) and autolysosomes (red spots) colocalization using the Image J software. Student’s *t* test. **O**, **P** KGN cells were treated with 10 μM CQ or DMSO for 4 h. Representative western blotting bands and the relative expression levels of LC3B and SQSTM1 were detected. (*n* = 3). one-way ANOVA. *p < 0.05; **p < 0.01; ***p < 0.001; ****p < 0.0001. *ns* not significant. The *P* value calculated by multiple comparison and one-way ANOVA test was corrected

We found an association between BOP1 and autophagy in patients with PCOS and animal models; thus, we evaluated the involvement of BOP1 in autophagy in PCOS pathogenesis. We knocked down BOP1 in KGN cells using a lentivirus, and the sequence with the highest knockdown efficiency was used in subsequent experiments (Fig. 5A, B). The western blotting results showed a significantly increased LC3-II/LC3-I ratio and Beclin1 levels and decreased SQSTM1 levels in the KGN cells when BOP1 was stably knocked down (Fig. 5C, D). Additionally, changes in autophagy flux in the KGN cells were analyzed by confocal microscopy, and the results showed significantly higher numbers of autophagosomes (yellow spots) and autophagolysosomes (red spots) in KGN cells of the LV-shBOP1 group than KGN cells of the LV-NC group (Fig. 5E, F). To further verify the changes in autophagy flux, we used CQ to block autophagic flow at the late lysosomal stage and detected the changes in autophagic proteins. The western blotting results showed that CQ treatment led to LC3-II accumulation in KGN cells in the LV-shBOP1 group (Fig. 5G, H), implying that autophagy was activated in the BOP1-knockdown KGN cells. To further verify the effect of BOP1 knockdown on autophagy in GCs, we established a KGN cell line stably overexpressing BOP1 (Fig. 5I, J) and a classical autophagy activation model using EBSS. The western blotting results showed that BOP1 overexpression decreased LC3-I-to-LC3-II conversion and increased SQSTM1 levels under EBSS-induced conditions (Fig. 5K, L). Additionally, autophagosome and autophagic lysosome formation in the KGN cells of the BOP1 overexpression group was lower than that in the KGN cells of the control group (Fig. 5M, N). CQ treatment further reduced autophagy flux in the BOP1 overexpression group (Fig. 5O, P). In summary, the above results suggest that BOP1 knockdown promotes autophagy activation in GCs.

### BOP1 regulates autophagy via the p53/mTOR pathway

**Fig. 6:**
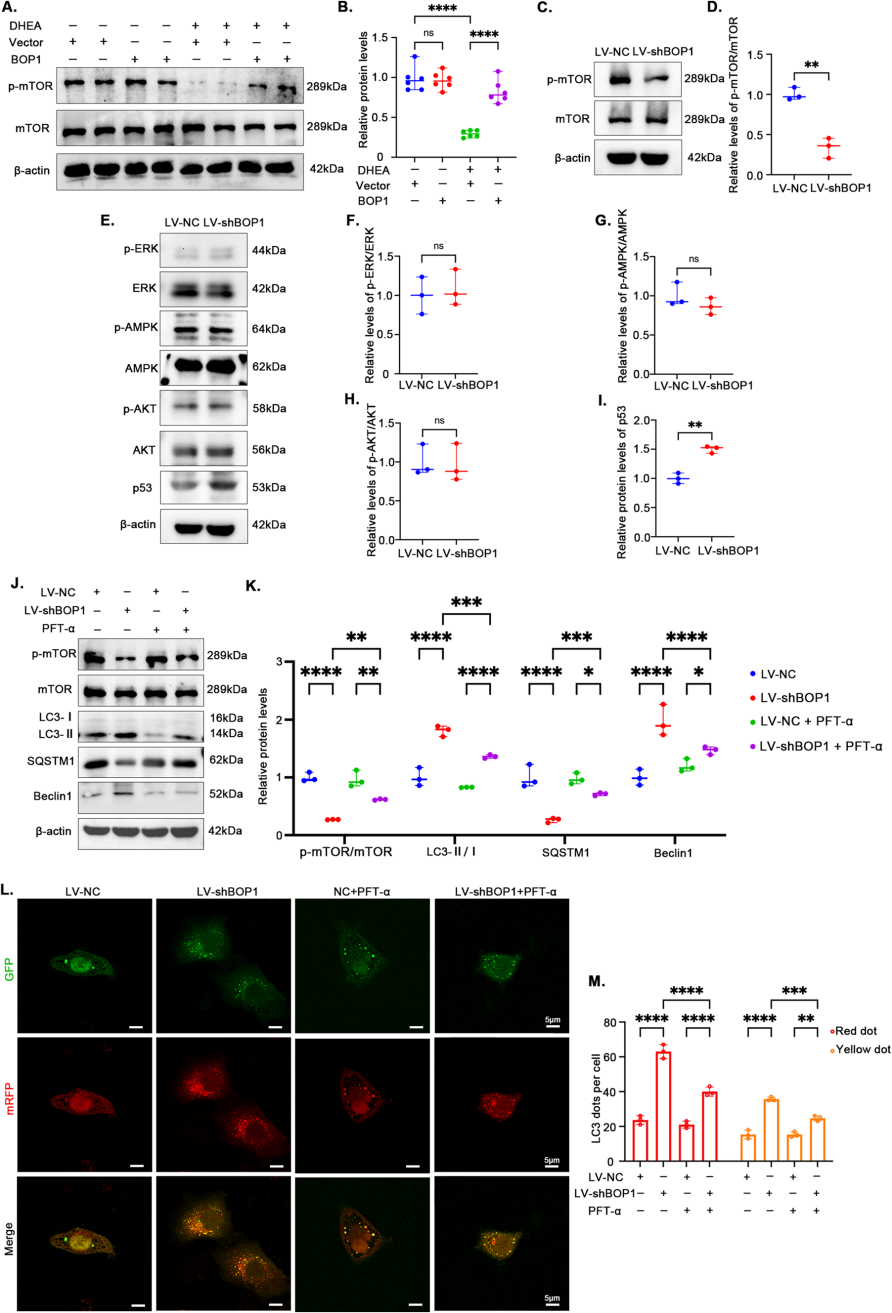
BOP1 regulates autophagy via p53/mTOR pathway. **A**, **B** Western blot and densitometric analysis for the expression of p-mTOR in the four groups (*n* = 3). one-way ANOVA. **C**, **D** Representative western blotting bands and the relative expression levels of p-mTOR in KGN cells infected with LV-NC or LV-shBOP1 (*n* = 3). Student’s *t* test. **E**–**I** Western blot and densitometric analysis for the expression of p-ERK, p-AMPK, p-AKT and p53 in KGN cells infected with LV-NC or LV-shBOP1 (*n* = 3). Student’s *t* test. **J**, **K** KGN cells were treated with 10 μM PFT-α or equal DMSO for 12 h, and protein levels of p-mTOR, LC3B, SQSTM1 and Beclin1 were detected with Western blot. one-way ANOVA. **L**, **M** Autophagic flux was assessed by quantification of autophagosomes (yellow spots) and autolysosomes (red spots) colocalization using the Image J software. one-way ANOVA. *p < 0.05; **p < 0.01; ***p < 0.001; ****p < 0.0001. *ns* not significant

mTOR is a key regulatory protein to initiate macroautophagy [24]. To investigate whether BOP1 initiated autophagy by regulating mTOR, we examined mTOR phosphorylation levels in mouse ovarian tissues. The western blotting results showed that these levels decreased significantly in the PCOS mice, indicating autophagy initiation. Conversely, LV-BOP1 intervention partially restored the mTOR phosphorylation levels (Fig. 6A, B). Additionally, mTOR phosphorylation was inhibited in KGN cells when BOP1 was silenced (Fig. 6C, D), suggesting that BOP1 regulated autophagy by modulating mTOR. Subsequently, we screened signaling pathways that might be associated with upstream mTOR signaling regulation. The western blotting results showed that ERK, AMPK, and Akt phosphorylation levels were unchanged in the KGN cells of the BOP1-knockdown group, whereas p53 levels were increased, suggesting that p53 might be involved in regulating autophagy in KGN cells (Figure E–I). To further verify the role of p53, we treated KGN cells with the p53 inhibitor PFT-α and probed the level of autophagy activation. The western blotting results showed that PFT-α partially reversed the decreased mTOR phosphorylation levels and inhibited autophagy activation in the KGN cells of the BOP1-knockdown group (Fig. 6J, K). Additionally, the results of autophagic vesicle and lysosome assays showed autophagy flux inhibition caused by the p53 inhibitor in the KGN cells (Fig. 6L, M). The above results suggest that BOP1 downregulation inhibits mTOR phosphorylation by regulating p53 activation, which in turn activates autophagy.

### BOP1 downregulation improves p53 stability via nucleolus stress responses

**Fig. 7:**
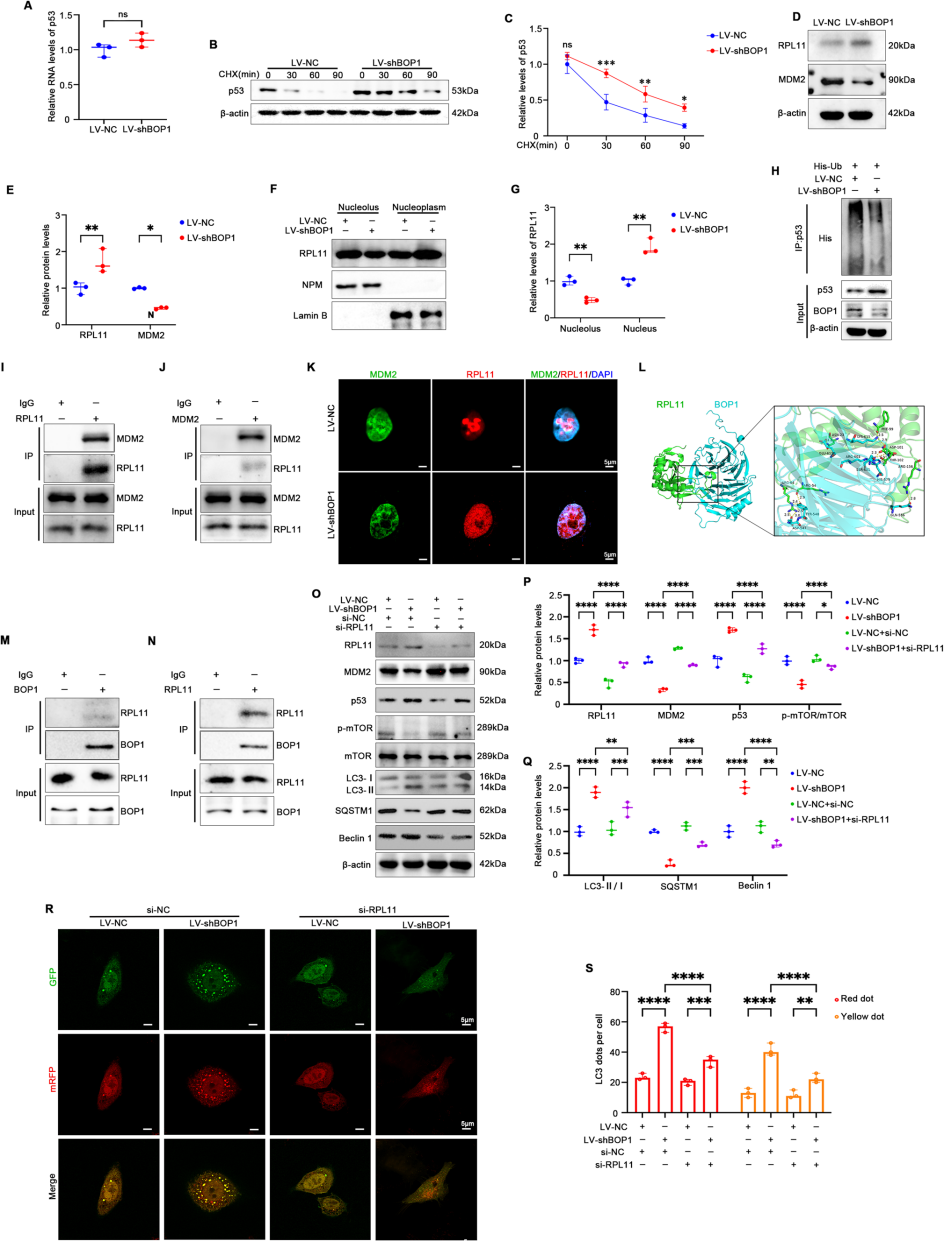
BOP1 downregulation enhances p53 stability through nucleolus stress response. **A** Quantification of the mRNA level of p53 in KGN cells infected with LV-NC and LV-shBOP1 (*n* = 3). Student’s *t* test. **B**, **C** KGN cells were transfected with LV-NC or LV-shBOP1 and administrated with CHX at different time for detecting the p53 degeneration rate by western blot (*n* = 3). **D**, **E** Western blot and densitometric analysis for the expression of RPL11 and MDM2 in KGN cells (*n* = 3). Student’s *t* test. **F**, **G** Western blot and densitometric analysis for the expression of RPL11 in the Nucleolus and nucleoplasm of KGN cells. Lamin B, and NPM are used as the nucleoplasm and Nucleolus markers (*n* = 3). **H** Effects of BOP1 knockdown on the MDM2-mediated p53 ubiquitination in KGN cells (*n* = 3). **I** Interaction between RPL11 and MDM2 in KGN cells determined by coimmunoprecipitation analysis using rabbit IgG or anti-RPL11 antibodies (*n* = 3). **J** Interaction between RPL11 and MDM2 in KGN cells determined by coimmunoprecipitation analysis using rabbit IgG or anti-MDM2 antibodies (*n* = 3). **K** Confocal microscopy was used to detect the location of RPL11 (Red) and MDM2 (Green) in KGN cells (*n* = 3). **L** Representative images of the docking mode of BOP1 binding to RPL11. **M** Interaction between BOP1 and RPL11 in KGN cells determined by coimmunoprecipitation analysis using rabbit IgG or anti-BOP1 antibodies (*n* = 3). **N** Interaction between BOP1 and RPL11 in KGN cells determined by coimmunoprecipitation analysis using rabbit IgG or anti-RPL11 antibodies. **O**–**Q**. Western blot and densitometric analysis for the expression of RPL11, MDM2, p53, p-mTOR, LC3B, SQSTM1, Beclin1 (*n* = 3). one-way ANOVA. **R**, **S.** Autophagic flux was assessed by quantification of autophagosomes (yellow spots) and autolysosomes (red spots) colocalization using the Image J software (*n* = 3). one-way ANOVA. *p < 0.05; **p < 0.01; ***p < 0.001; ****p < 0.0001. *ns* not significant

BOP1 acts as a ribosome biosynthesis factor, and alterations in its protein level may affect ribosome synthesis, thus ultimately leading to nucleolus stress [25]. To explore how BOP1 regulates p53, we examined p53 mRNA levels. The results showed that p53 mRNA levels were not significantly altered in the BOP1 knockdown compared with those in the control group, indicating that BOP1 downregulation did not affect the transcriptional regulation of p53 (Fig. 7A). We subsequently treated KGN cells with CHX, and the western blotting results showed that BOP1 silencing resulted in the prolonged half-life of p53 (Fig. 7B, C). The disruption of ribosome biosynthesis leads to nucleolus stress, which triggers the p53 signaling pathway via RPL11 [26]. Therefore, we examined RPL11 and MDM2 levels in KGN cells and found that GCs in the BOP1-knockdown group showed increased RPL11 levels and decreased MDM2 levels (Fig. 7D, E). Next, we examined the protein levels of rpl11 in the nucleolus and nucleoplasm. It was found that BOP1 silencing decreased the protein level of RPL11 in the nucleolus and increased the protein level of RPL11 in the nucleoplasm (Fig. 7F, G), which suggests that knockdown of BOP1 prompted the release of RPL11 from the nucleolus to the nucleoplasm. When nucleolus stress occurs, RPL11, a ribosome synthesizing factor, is released from the nucleolus into the nucleoplasm, where it binds to MDM2, thereby inhibiting MDM2-mediated p53 ubiquitination [19]. Our results showed that the level of ubiquitination of P53 in KGN cells was reduced after BOP1 knockdown. (Fig. 7H). Besides, the CO-IP results and the immunofluorescence assay showed that RPL11 was mainly distributed in the nucleolus in the control cells, whereas it was distributed in the nucleoplasm and co-localized with MDM2 in the BOP1-silenced cells (Fig. 7I-K). The above results suggest that BOP1 downregulation may inhibit the E3-ubiquitin ligase MDM2 by activating RPL11, thus improving p53 stability. To verify the interaction between BOP1 and RPL11, we first modeled the possible interaction sites of BOP1 and RPL11 by molecular docking (Fig. 7L). The CO-IP results showed an interaction between BOP1 and RPL11 in KGN cells (Fig. 7M, N). To further verify whether BOP1 regulated the p53 pathway via nucleolus stress responses, we used RPL11 siRNA to transfect KGN cells, and the western blotting results showed that RPL11 knockdown reversed p53 activation via BOP1, which ultimately blocked autophagy activation in KGN cells (Fig. 7O–Q). Similarly, the autophagy flux assay results showed that RPL11 knockdown inhibited autophagosome and autophagy-lysosome generation in KGN cells with stable low BOP1 expression (Fig. 7R, S). In summary, these results suggest that BOP1 activates the p53 signaling pathway via the nucleolus stress response, thereby promoting aberrant autophagy in KGN cells.

### Correlation between BOP1 and PCOS development

**Fig. 8:**
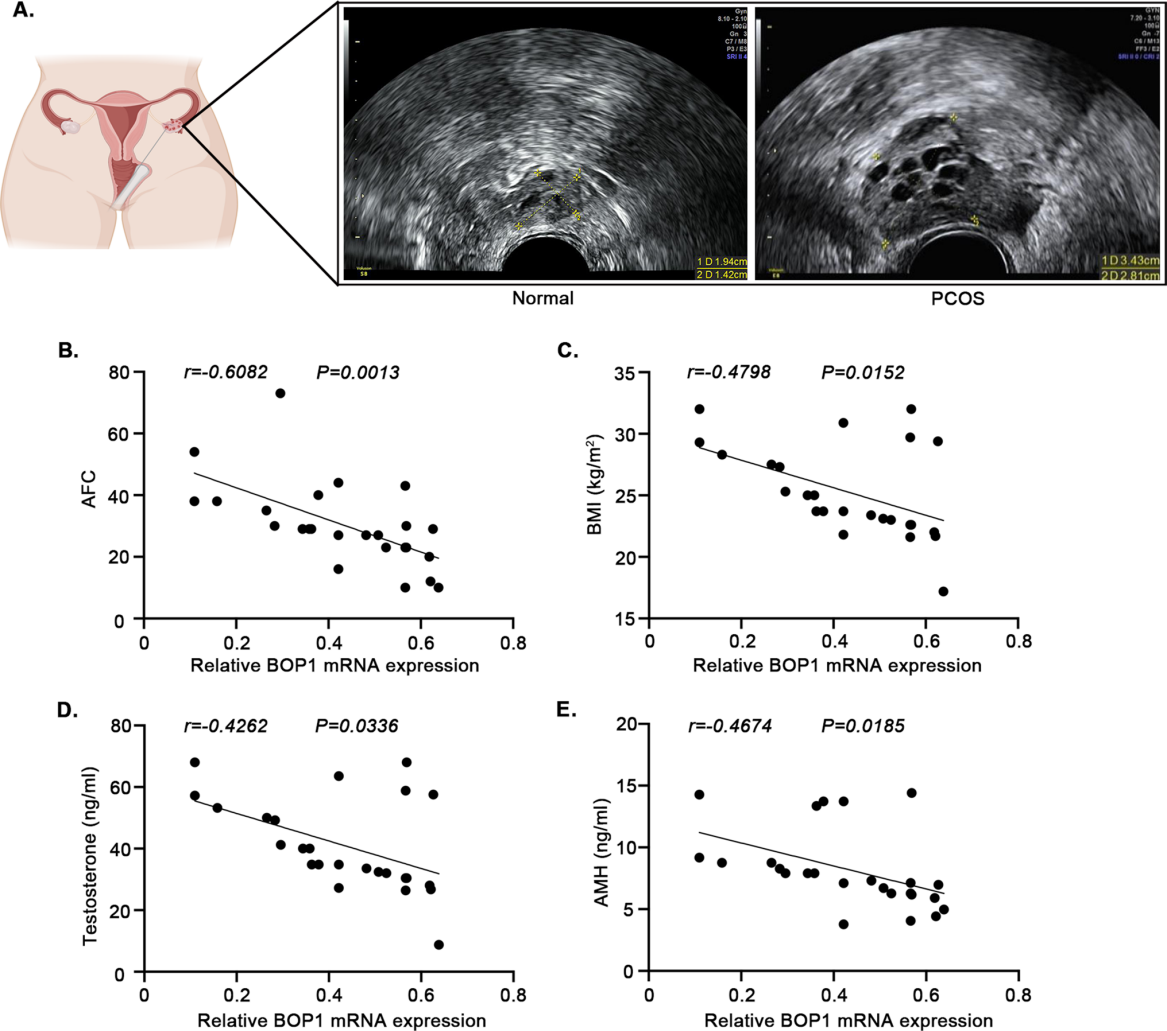
Correlation between BOP1 and the development of PCOS. **A** Transvaginal ultrasound in patients with PCOS and healthy control women. **B**–**E** Association of BOP1 expression with AFC, BMI, Testosterone, and AMH

To investigate the potential value of BOP1 in PCOS, we evaluated the correlation between BOP1 mRNA levels in the GCs of patients with PCOS and their clinical indicators. The results showed that in these patients, decreased BOP1 mRNA levels in ovarian GCs were negatively correlated with increased antral follicle count (AFC), body–mass index (BMI), serum androgen levels, and anti-Mullerian hormone (AMH) (Fig. 8). The above results suggest that BOP1 may play a crucial role in PCOS pathogenesis.

## Discussion

The specific pathogenesis of PCOS has not been elucidated owing to its highly heterogeneous nature. This is the first study to reveal the potential role of BOP1 in PCOS. BOP1 downregulation activates aberrant autophagy in GCs, contributing to PCOS initiation and progression via the nucleolar stress signaling pathway.

The GC is an essential component of follicles that produces follicle-stimulating hormone and estrogen, ensures orderly oocyte growth and development via bidirectional communication, and precisely regulates meiotic arrest and oocyte division resumption [27]. Therefore, the maintenance of the normal physiologic activity of GCs is critical for female reproductive and endocrine homeostasis. Previous studies have shown that autophagy levels in GCs increase abnormally in patients with PCOS. Abnormal autophagy activation decreases GC levels, thus affecting oocyte development and maturation and leading to ovulatory dysfunction [28]. Herein, we investigated the GCs of patients with PCOS and animal models and demonstrated autophagy activation in GCs, which was consistent with earlier studies.

BOP1, a member of the PeBoW complex, is localized to the nucleolus and regulates ribosomal RNA synthesis. Its knockdown or mutation affects ribosomal RNA maturation and normal cell cycle order [29]. Previous studies on BOP1 have mainly focused on its role in cancer [13, 30]. Herein, based on the mRNA sequencing result that BOP1 expression was downregulated in the ovarian tissues of existing PCOS animals, we preliminarily verified the presence of the same alteration in GCs of patients with PCOS and the ovarian tissues of PCOS rats. To investigate the potential involvement of BOP1 in PCOS, we established a BOP1-overexpression animal model and subsequently found that BOP1 upregulation improved the phenotypes of hyperandrogenism, follicular dysplasia, and disrupted estrus cycle of the PCOS mice and ameliorated the abnormal autophagy in the mice. Further, autophagy activation was confirmed to occur in KGN cell lines with the stable low expression of BOP1, suggesting that the mechanism by which BOP1 was involved in PCOS development was related to autophagy regulation.

Autophagy is a conserved mechanism for self-degradation to recycle and reuse cellular cargo [7]. It is a protective mechanism triggered during cellular stress such as energy and oxidative stresses, and moderate autophagy helps maintain cellular physiological homeostasis. However, abnormal autophagy in pathological states affects cellular energetic and functional homeostasis and exacerbates disease progression. For instance, HMGB1 upregulation promotes IR by promoting autophagy in PCOS GCs [31]. mTOR, a prominent regulatory protein in cellular energy metabolism, is activated under physiological energy conditions to promote rRNA synthesis and protein synthesis, which ensure normal cell growth [32]. Under cellular stresses, mTOR activity is inhibited and autophagy is induced to maintain cellular metabolic homeostasis [33].

We observed reduced mTOR phosphorylation levels and autophagy activation in the PCOS model mice, whereas BOP1 overexpression partially reversed mTOR activity inhibition. Additionally, mTOR inhibition was detected in a BOP1 knockdown KGN cell line, indicating that autophagy was activated by BOP1 downregulation via mTOR. Numerous regulators upstream of mTOR, such as PI3K/AKT, AMPK, ERK, and p53, can regulate the expression of downstream autophagy proteins in several diseases by activating or inhibiting mTOR [34–37]. To investigate the mechanism by which BOP1 affects autophagy, we examined the levels of the mentioned proteins in KGN cells and found that BOP1 silencing increased p53 levels significantly in KGN cells, whereas PI3K/AKT, AMPK, and ERK levels were unaffected. Additionally, a P53 inhibitor partially blocked autophagy activation in BOP1-knockdown KGN cells. Thus, BOP1 inhibits mTOR activity via p53, thereby activating autophagy in GCs.

The nucleolus is a highly active subcellular organelle where ribosomes are formed. When ribosome biogenesis is disrupted, a stimulus generated is sensed by the nucleolus. The organelle responds to it via nucleolar stress, which usually involves the classical p53 signaling pathway [38]. Under physiological conditions, p53 is degraded via the ubiquitin–proteasome pathway mediated by the E3-ubiquitin ligase MDM2 to maintain its low intracellular level. When nucleolar stress is initiated, ribosomal proteins are released from the nucleolus into the nucleoplasm and subsequently bind to the E3-ubiquitin ligase MDM2 to inhibit MDM2-mediated p53 ubiquitination, thus leading to p53 accumulation, and ultimately triggering a series of downstream events such as apoptosis, cell-cycle arrest, senescence, and genotoxic stress [39]. Recently, the potential association between nucleolar stress and autophagy has been explored, and the effect of nucleolar stress on autophagy is garnering worldwide attention. In colon cancer studies, the decreased level of the ribosomal protein universally conserved ribosomal protein L3 induces autophagy activation via the nucleolar stress pathway, thereby causing chemoresistance [40]. As an important participant in ribosome biogenesis, BOP1 downregulation may exhibit a similar core mechanism in autophagy activation in PCOS animal models. We determined p53 accumulation in BOP1-knockdown KGN cells by assaying its RNA and protein levels and subsequently confirmed that BOP1 downregulation induced nucleolar stress by RPL11 and MDM2 levels. The protein interactions between BOP1 and RPL11 in the KGN cells were determined by molecular docking and CO-IP, and the results showed that the RPL11 siRNA intervention inhibited autophagy activation in the KGN cells via BOP1 knockdown. This result indicated that BOP1 activated autophagy in GCs in PCOS via the p53-dependent nucleolus stress response.

The presence of clinical or biochemical manifestations of hyperandrogenemia is one of the diagnostic indicators of PCOS, whereas another typical clinical feature is polycystic changes in ovaries that can be detected by ultrasound. Additionally, a majority of patients are accompanied by excessive weight gain and increased AMH levels [41]. The analysis of clinical indicators in patients with PCOS showed that BOP1 levels were substantially associated with the AFC, BMI, serum androgen level, and AMH level of the patients, which suggested that BOP1 could be a potential therapeutic target for PCOS. In summary, our data suggest that BOP1 holds the potential to serve as an indicator of adverse clinical phenotypes in PCOS patients, contributing to personalized diagnosis and treatment. Further, the development of drugs such as locally targeted ovarian modulation of BOP1 or inhibition of kernel stress may help alleviate the symptoms of hormonal abnormalities and follicular dysplasia and improve the quality of life and pregnancy dilemma of PCOS patients.

The present study has some limitations. The autophagy levels of different organs or cells were found to vary in patients with PCOS and animal models [8]. Additionally, we focused on the mechanism by which BOP1 affects autophagy in GCs, and the results would be more convincing if BOP1 could be specifically overexpressed in mouse ovarian GCs. Further, the regulatory effect of p53 on autophagy is related to its localization in the cell. We focused on exploring the mechanism by which p53 regulates mTOR in the cytoplasm and then drives autophagy. However, p53 in the nucleus acted as a transcription factor to induce the expression of the autophagy related protein, thus directly driving autophagy [42]. Moreover, p53 can directly bind to the autophagy regulator to directly participate in autophagosome formation [43]. Therefore, BOP1 downregulation resulting in p53 accumulation probably activates downstream autophagy by regulating autophagy protein transcription or directly participating in autophagosome synthesis. The mouse model of PCOS, which is mainly induced by the administration of hyperandrogenism, has clear pathogenic factors and phenotypes, whereas the etiology of PCOS patients is unknown, and their clinical manifestations vary depending on the environment, ethnicity, and genetics [44]. Besides, although BOP1 is a highly conserved gene within mammals, it may still differ between humans and mice. Therefore, the corresponding regulatory mechanism of BOP1 extracted from mice with PCOS may not accurately represent its possible role in patients with PCOS, and the potential role of BOP1 in the pathophysiological process of PCOS still requires further investigation and characterization.

**Fig. 9:**
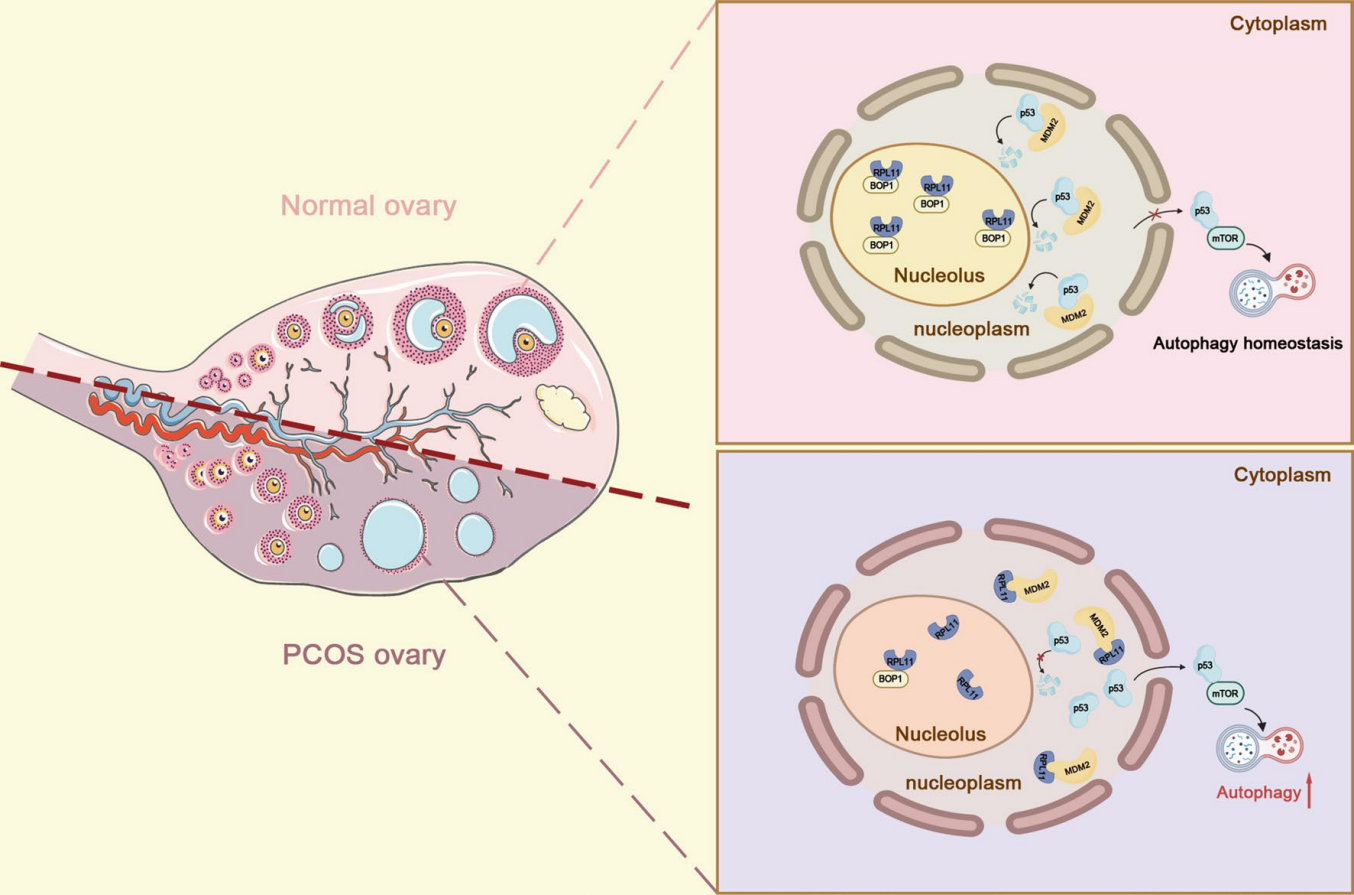
Graphics summarizes the mechanism of BOP1 knockdown in regulation of autophagy in PCOS. BOP1 knockdown enhanced the binding of RPL11 and MDM2, hence causing p53 activation. Therefore, p53 inhibited the phosphorylation of mTOR, thereby activating autophagy in GCs. *BOP1* Block of proliferation, *PCOS* polycystic ovary syndrome, *GC* granulosa cell, *RPL11* ribosomal protein L11, *MDM2* murine double minute 2, *mTOR* mammalian target of rapamycin

In conclusion, we elucidated the role of BOP1 in the aberrant activation of autophagy in PCOS GCs for the first time. BOP1 knockdown activates GC autophagy via a p53-dependent nucleolar stress response (Fig. 9). Therefore, BOP1 may be a novel molecular target to ameliorate abnormal autophagy in PCOS GCs. The present findings provide new perspectives and a basis to further investigate PCOS pathogenesis.

### Supplementary Information

Below is the link to the electronic supplementary material.

Supplementary file1 (DOCX 15 KB)

Supplementary file2 (DOCX 13 KB)

## Data Availability

The datasets used during the current study are available from the corresponding author on reasonable request.
