# Supplementary material for: BOP1 contributes to the activation of autophagy in polycystic ovary syndrome via nucleolar stress response
Source: Cell Mol Life Sci. 2024 Feb 27;81(1):101. doi: 10.1007/s00018-023-05091-1 (PMC10896891; doi:10.1007/s00018-023-05091-1)
Supplement: Supplementary file 1 — Supplementary file1 (DOCX 15 KB) [file 18_2023_5091_MOESM1_ESM.docx]

Clinical features of recruited participants

| Clinical features | PCOS group | Non-PCOS group | P values |
| --- | --- | --- | --- |
| n | 197 | 366 | — |
| Age (year) | 29.56±3.68 | 30.54±3.01 | **0.001** |
| Height (cm) | 160.35±4.82 | 160.25±4.84 | 0.828 |
| Body weight (kg) | 62.21±8.54 | 54.45±7.03 | **＜0.001** |
| BMI (kg/m^2^) | 24.18±3.16 | 21.17±2.35 | **＜0.001** |
| Basal FSH | 6.79±2.03 | 7.55±2.38 | **＜0.001** |
| Basal E2 | 52.14±20.15 | 51.28±24.26 | 0.752 |
| Basal P | 0.86±0.03 | 0.69±0.07 | 0.141 |
| Basal PRL | 11.34±6.03 | 14.24±7.13 | **＜0.001** |
| Basal LH | 8.83±4.78 | 4.42±2.55 | **＜0.001** |
| Basal T | 34.50±5.16 | 23.08±10.04 | **＜0.001** |
| AMH | 7.71±3.42 | 3.85±2.42 | **＜0.001** |
| LH/FSH | 1.25±0.81 | 0.61±0.39 | **＜0.001** |
