# Supplementary material for: BOP1 contributes to the activation of autophagy in polycystic ovary syndrome via nucleolar stress response
Source: Cell Mol Life Sci. 2024 Feb 27;81(1):101. doi: 10.1007/s00018-023-05091-1 (PMC10896891; doi:10.1007/s00018-023-05091-1)
Supplement: Supplementary file 2 — Supplementary file2 (DOCX 13 KB) [file 18_2023_5091_MOESM2_ESM.docx]

The sequences of the primers for qRT-PCR.

| Gene | Primer Sequence (5–3′) |
| --- | --- |
| BOP1 | GCCACAAGATGCACGTACCT |
|  | TTCCTGGATGAAGCGTCCGTA |
| p53 | CTCACCATCATCACACTGGAA |
|  | TCATTCAGCTCTCGG AACATC |
| β-actin | CATGTACGTTGCTATCCAGGC |
|  | CTCCTTAATGTCACGCACGAT |
